# Diurnal Fe(II)/Fe(III) cycling and enhanced O_2_ production in a simulated Archean marine oxygen oasis

**DOI:** 10.1038/s41467-021-22258-1

**Published:** 2021-04-06

**Authors:** A. J. Herrmann, J. Sorwat, J. M. Byrne, N. Frankenberg-Dinkel, M. M. Gehringer

**Affiliations:** 1 Department of Microbiology, Technische Universität Kaiserslautern, 67663 Kaiserslautern, Germany; 2 Geomicrobiology, Centre for Applied Geosciences, University of Tübingen, 72074 Tübingen, Germany

**Keywords:** Environmental microbiology, Biogeochemistry

## Abstract

The oxygenation of early Earth’s atmosphere during the Great Oxidation Event, is generally accepted to have been caused by oceanic Cyanobacterial oxygenic photosynthesis. Recent studies suggest that Fe(II) toxicity delayed the Cyanobacterial expansion necessary for the GOE. This study investigates the effects of Fe(II) on two Cyanobacteria, *Pseudanabaena* sp. PCC7367 and *Synechococcus* sp. PCC7336, in a simulated shallow-water marine Archean environment. A similar Fe(II) toxicity response was observed as reported for closed batch cultures. This toxicity was not observed in cultures provided with continuous gaseous exchange that showed significantly shorter doubling times than the closed-culture system, even with repeated nocturnal addition of Fe(II) for 12 days. The green rust (GR) formed under high Fe(II) conditions, was not found to be directly toxic to *Pseudanabaena* sp. PCC7367. In summary, we present evidence of diurnal Fe cycling in a simulated shallow-water marine environment for two ancestral strains of Cyanobacteria, with increased O_2_ production under anoxic conditions.

## Abstract (web summary)

Cyanobacterial photosynthesis is thought to have oxygenated Earth’s atmosphere during the Great Oxidation Event, but these organisms had to overcome the toxic effects of iron. Here the authors simulate Archaean conditions in Cyanobacterial cultures and find that gas exchange and rust formation alleviated iron toxicity.

## Introduction

**Fig. 1:**
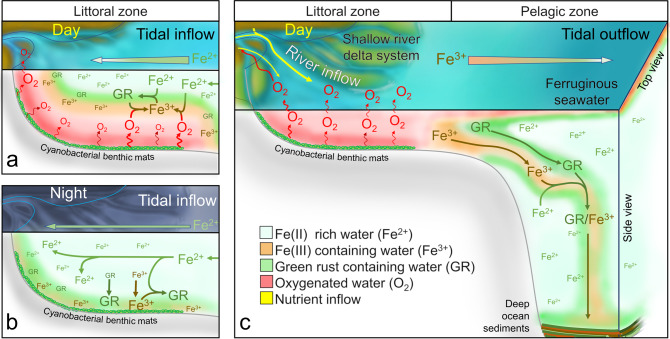
Graphical model of a proposed Archaean littoral oxygen oasis. **a** A diurnal tidal inflow of ferruginous seawater creates an anoxic Fe(II) layer over oxygenic phototrophic Cyanobacterial mats. Photosynthetic oxygen creates green rust (GR) in the upper boundary layer by partial oxidation of Fe(II) during the day. Closer to the Cyanobacterial mats, Fe(III) is the dominant species, as Fe(II) and precipitating GR is completely oxidised. **b** During the night time, no oxygen is produced by photosynthesis and the littoral zone becomes ferruginous with precipitated Fe(III) oxides and/or GR, produced in the daytime, at the bottom layer. **c** Tidal outflow transports GR and Fe(III) in the anoxic open ocean, where the remaining Fe(III) can react with Fe(II) to GR. GR and/or Fe(III) sink to the bottom and form deep ocean sediments. High oxygen levels during the day oxidise Fe(II) from the pelagic zone and thereby prevent Fe(II) from reaching the littoral zone. Surplus oxygen not consumed by the oxidation of Fe(II) escapes into the atmosphere leading to localised land weathering, thereby releasing additional nutrients. Additional weathering at the oxygen-rich microbe–mineral interface cannot be excluded. Image created from references^6, 12, 13, 17, 18^.

The start of the Great Oxidation Event (GOE) ~2.43 Ga (billion years ago)^1^ marked the beginning of the Proterozoic Eon and initiated the permanent oxygenation of Earth’s atmosphere and oceans^2^. Although the GOE is associated with the widespread release of oxygen presumably by oxygenic phototrophic Cyanobacteria^3^, the rock record shows indications of oxygenic photosynthesis 200 Myr (million years) prior, in the form of tufted microbial mats and stromatolites in the 2.7 Ga Tumbiana Formation, West Australia^4, 5^. Potential indications of phototrophic mats with Cyanobacterial tufted mat morphology have been identified in the widespread shallow tidal mats in the 3.2 Gyr (billion years) old Moodies Group, South Africa, which are thought to be phototrophic^6, 7^. Geochemical markers that reflect transient or local occurrences of low levels of oxygen are also indicators for oxygenic photosynthesis prior to the GOE at 2.8–3.0 Gyr^8, 9^. Many hypotheses exist as to why the occurrence of oxygenic photosynthesis and the timing of the GOE are temporally uncoupled, including the consumption of released oxygen by redox-sensitive elements, such as sulphur, or cell respiration^3^, the time needed to adapt to a pelagic life style necessary to produce O_2_ on a large scale^10, 11^ or the toxicity of an anoxic, ferruginous Archaean ocean^12^. All of these proposed processes may have contributed towards restricting the expansion of Cyanobacteria to a few highly productive niches, thereby generating the occasional ‘oxygen oases’ recorded in the rock record^13, 14^. These localised hot-spots bearing up to 10 µM dissolved oxygen^14^ would have mitigated the reported toxic effects of Fe(II) due to oxidation to insoluble Fe(III)^12^. Fe(III) in turn could have played a major role in the formation of banded iron formations (BIF), either directly as Fe(III) oxides^15, 16^ or as green rust (GR), after reacting with locally remaining Fe(II)^17^. The localised concentration of oxygenic phototrophs may have resulted in weathering of nearby rock surfaces, releasing essential micronutrients, such as sulphates and molybdenum^18^. By combining these hypotheses, a graphical representation of an Archaean oxygen oasis was created (Fig. 1) to illustrate diurnal Fe(II)/(III) fluxes.

Simulations of the processes related to Archaean ‘oxygen oases’ under today’s atmosphere in small-scale laboratory conditions remain challenging and are conducted either in ‘closed’ bottle systems^12, 19^ or ‘open’ anoxic workstations^20^. Differences in experimental setups and Cyanobacterial strains used make it difficult to obtain a clear overview of Fe(II) toxicity and its effects on photosynthesis in ancestral Cyanobacterial strains. Cyanobacteria are the only bacteria that possess a circadian clock that regulates, amongst others, iron uptake and the redox status within the cell^21^, making it essential to conduct experiments on Archaean Fe(II) toxicity under diurnal conditions. Here we directly compare two culture systems by setting up otherwise identical experiments to determine the reported toxicity of Fe(II) on two basal clade, saltwater Cyanobacteria strains, the multicellular *Pseudanabaena* sp. PCC7367 and unicellular *Synechococcus* PCC7336. The simpler closed-culture system utilises a sealed container with a static initial atmosphere with no gaseous exchange. The open-culture system permits continual gaseous exchange with the larger atmospheric reservoir of an anoxic workstation, which removes O_2_ and replenishes CO_2_ in the atmosphere. The strains were chosen because they are the most-primitive recent saltwater Cyanobacteria, with a close phylogenetic relationship to the root Cyanobacteria, *Gloeobacter violaceus* sp. PCC7421^11, 22^. The collected data for cell growth, Fe(II) oxidation and O_2_ production rates not only provide important constraints for further theoretical modelling, but also show the shortcomings of a closed growth system. After establishing an appropriate experimental setup for Fe(II) toxicity experiments, we explored the influence that repeated nocturnal addition of Fe(II) has on Cyanobacterial growth, in contrast to the single exposure experiments conducted so far. Of particular interest was the observation of GR formation during periods of low photosynthetic activity, when Fe(II) concentrations were high, as GR is thought to be an important contributor to the formation of BIF^17^.

## Results

### Iron toxicity in different growth systems

To assess Fe(II) toxicity and the influence of the experimental setup on growth, two marine basal clade Cyanobacteria, *Pseudanabaena* sp. PCC7367 and *Synechococcus* sp. PCC7336, were grown for 21 days with different Fe(II) starting concentrations approximating the proposed range of an Archaean ocean of 30–120 µM^23^. The growth systems were either closed bottles with a single injection of nitrogen with 10% CO_2_ and 5% H_2_ at the start of the experiment or an open system with ventilated culture flasks inside an anoxic chamber filled with forming gas (N_2_ with ≤ 5% H_2_) and constant removal of O_2_ while maintaining 0.2% of CO_2_, which is on the lower end of the proposed CO_2_ concentrations of an Archaean atmosphere^24, 25^. Oxic control cultures were grown in parallel under normal atmospheric conditions in the open-culture system for comparison. The growth medium was buffered with 22 mM NaHCO_3_ to minimise the shift in media pH under different CO_2_ concentrations.

**Fig. 2:**
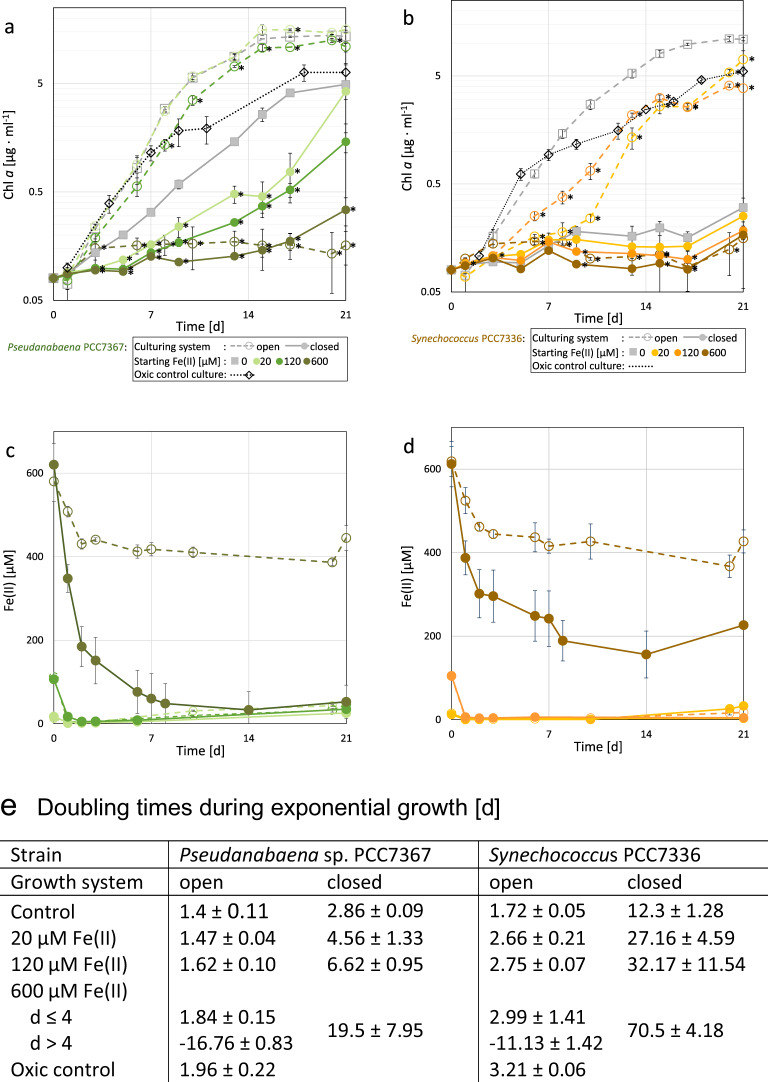
Illustration of the effects of the addition of different concentrations of Fe(II) on the growth of *Pseudanabaena* sp. PCC7367 and *Synechococcus* sp. 7336. Comparison of growth (**a**, **b**, **e**) and Fe(II) oxidation (**c**, **d**) of *Pseudanabaena* sp. PCC7367 (**a**, **c**) and *Synechococcus* sp. PCC7336 (**b**, **d**) between the open- and a closed-culture systems. The growth response of the two strains of saltwater Cyanobacteria in 22 mM NaHCO_3_-buffered ASN-III media to different concentrations of Fe(II) was measured by Chl *a* determination every 2–3 days (**a**, **b**) for 21 days in closed anoxic culture bottles (solid lines) or open cell suspension flasks in an anoxic workstation (dashed lines). The Fe(II) starting concentrations were 20 µM (PCC7367 light green/PCC7336 yellow), 120 µM (PCC7367 green/PCC7336 orange) and 600 µM (PCC7367 dark green/PCC7336 red). The controls (grey) were grown in ASN-III media with an Fe(III) concentration of 15 µM under anoxic (solid/dashed line) and normal atmospheric conditions (dotted line). The oxidation of Fe(II) to Fe(III) was assessed in parallel using a spectrophotometric assay (**c**, **d**). Fe(II) measurements of abiotic controls are depicted in Supplementary Fig. 6. Cell doubling times (**d**) and their standard deviations during the exponential growth phase (**e**) were determined from day 1 to 13 for *Pseudanabaena* sp. PCC7367 and from day 1 to 17 for *Synechococcus* sp. PCC7336. The doubling time for the cultures with 600 µM Fe(II) in the open-culture system was split into a doubling time before green rust formation at day 4 and after. Data are presented as mean values ± SD with *n* = 4 biologically independent samples. Asterisks (*) represent a significant difference to the media control grown in the equivalent growth system (*p* < 0.05, Student’s *t*-test, two-tailed).

Monitoring of chlorophyll *a* (Chl *a*) levels of photosynthetic organisms is a standard method for determining growth rates^26^. In the closed-culture system, *Pseudanabaena* sp. PCC7367 exhibits a significantly reduced Chl *a* growth if Fe(II) is added at the start, with a doubling time of up to ~20 days in 600 µM Fe(II) (Fig. 2a, e). In contrast, *Pseudanabaena* sp. PCC7367 in the anoxic open-culture system shows no significant difference in growth between the media controls and the cultures with 20 µM Fe(II). Incubation with 120 µM Fe(II) resulted in a small, but significant, delay in Chl *a* accumulation. Regardless of the culture system used, Fe(II) supplied at concentrations of 120 µM or less was completely oxidised within 2 days (Fig. 2c). Oxidation of 600 µM Fe(II), however, took up to 14 days in the closed-culture system, whereas in the open-culture system growth (Fig. 2a, b) Fe(II) oxidation (Fig. 2c, d) ceased after day 4. The slight increase in measured media Fe(II) at the end of stationary phase is most likely an artefact created by the incomplete removal of cell debris during the short centrifugation step to prevent Fe(II) oxidation during sample handling.

In the closed-culture system, *Synechococcus* PCC7336 demonstrated almost no growth, with a doubling time of ~12 days for the control and more than double that upon addition of Fe(II) up to 20 or 120 µM (Fig. 2b, e). In the cultures provided with 600 µM Fe(II) hardly any Chl *a* growth was observed (doubling time of >70 days). Also, the oxidation of Fe(II) ceased after day 7, indicating low biological activity (Fig. 2d). In the anoxic open-culture system *Synechococcus* PCC7336 control cultures grew rapidly, with a Chl *a* growth approaching those of *Pseudanabaena* sp. PCC7367 anoxic control cultures (Fig. 2e). However, addition of Fe(II) up to 20 and 120 µM increased the doubling times significantly (*p* < 0.05). Irrespective of which culture system was used, Fe(II) was completely oxidised after 2 days in cultures exposed to 20 and 120 µM of Fe(II) (Fig. 2d). Cultures of *Synechococcus* sp. PCC7336 grown with 600 µM Fe(II) in the open system showed a similar response to that seen for *Pseudanabaena* sp. PCC7367, with a cessation of biological activity after day 4 (Fig. 2b, d). At this point, the green precipitate, forming in the culture flasks of both strains, was collected and analysed by ^57^Fe Mössbauer spectroscopy, which identified the substance as GR (Supplementary Fig. 7 and Supplementary Table 4).

In summary, the data show a highly significant (*p* < 0.005) decrease in Chl *a* growth for both strains in the closed-culture vessels in comparison to the open-culture system at Fe(II) levels representing the Archaean ocean or lower (Fig. 2a, b, e). The open-culture system controls show no significant difference in Chl *a* growth to the present atmosphere controls for the first 10 days, after which the open-culture system shows better growth (Fig. 2a, b).

### Iron toxicity at daily exposure

To further expand on the experiments described above, which demonstrated that all Fe(II) in the concentration range proposed to have existed in the Archaean ocean was oxidised within 2 days, a second set of experiments were conducted to assess the effects of repeated exposure to Fe(II). In order to simulate a hypothetical reoccurring inflow of ferruginous Archaean seawater, Fe(II) was added to a concentration of ~120 µM every dark cycle for 12 days. The addition of Fe(II) was conducted once the O_2_ levels had dropped down close to zero due to cell respiration and passive gas exchange with the anoxic atmosphere in order to maximise the effect of Fe(II) exposure.

**Fig. 3:**
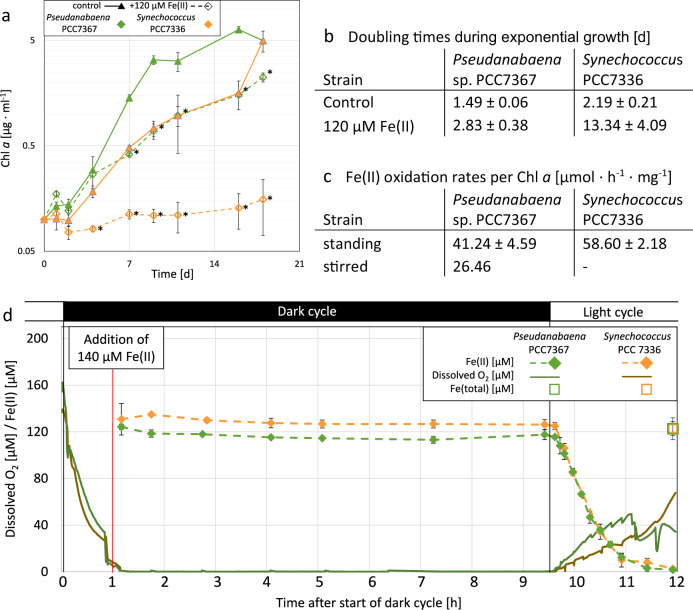
The influence of reoccurring nightly addition of Fe(II) on the growth of *Pseudanabaena* sp. PCC7367 and *Synechococcus* sp. PCC7336. Both species were cultured in an open-culture system (**a**, **b**) and the oxidation of Fe(II) monitored (**c**, **d**). Chl *a* growth curves (**a**) and doubling times (**b**) of the cultures in an anoxic open-culture system with a recurring nightly addition of 140 µM Fe(II). For the first 12 days during every dark cycle, the cultures were shaken briefly to release the dissolved oxygen and Fe(II) solution was added to a total concentration of 120–140 µM. Chl *a* measurements (**a**) were taken every 2nd to 3rd day for 19 days. Cultures grown without Fe(II) addition were used as controls (solid line). Cell doubling times during the exponential growth (**b**) were determined from day 2 to 12 for both strains. The retention of Fe(II) during the dark cycle (**d**) was exemplary tested in analogous 7-day old, exponentially growing control cultures of *Pseudanabaena* sp. PCC7367 (green) and *Synechococcus* PCC7336 (orange) by measuring the Fe(II) concentration (dashed lines), while in parallel recording the oxygen concentration (solid line). The Fe(II) oxidation was measured every 10 min with a spectrophotometric ferrozine assay. Two hours after the start of the light cycle, the amount of Fe(III) in the media was assessed (open squares). The average rate of Fe(II) oxidation per Chl *a* during the light cycle (**c**) was calculated and compared to cultures which were additionally stirred at constant 250 rpm on a magnetic stirrer. Data are presented as mean values ± SD with *n* = 4 biologically independent samples, except for the stirred Fe(II) oxidation rate with *n* = 2 biologically independent samples. Asterisks (^*^) represent a significant difference between Fe(II) supplemented culture and control (*p* < 0.05, Student’s *t*-test, two-tailed).

Repeated exposure to Fe(II) during the dark cycle over 21 days caused a significant (*p* < 0.05) drop in growth in both strains (Fig. 3a, b). Daily addition of Fe(II) leads to an increase in the doubling time of *Pseudanabaena* sp. PCC7367 by ~50%. *Synechococcus* PCC7336 was more sensitive to the prolonged Fe(II) exposure with a ~4x longer doubling time than the control culture. After day 4, the addition of Fe(II) during the dark cycle led to the temporary formation of a green precipitate, with an average Fe(II)/Fe(III) ratio of 2.54 ± 0.025, as determined by ferrozine assay for PCC7336, which is indicative of GR. During the first few hours of the light cycle, the precipitate was oxidised to a yellow/orange solid in the cultures of *Pseudanabaena* sp. PCC7367, while the oxidation took several days in the cultures of *Synechococcus* sp. PCC7336.

The Fe(II) retention experiment (Fig. 3d) showed that, in the absence of oxygenic photosynthesis, no Fe(II) oxidation occurs and cultures undergoing repeated Fe(II) exposure (Fig. 3a) were exposed to Fe(II) during the whole night cycle. The rapid decrease in dissolved Fe(II) levels, accompanied by an increase in the dissolved oxygen concentration, is only measurable after the start of the light cycle. The total iron levels measured 2 h after the start of the light cycle showed that almost all Fe(II) was oxidised. Repeating this experiment with stirred cultures of *Pseudanabaena* sp. PCC7367 demonstrated that even with increased release of oxygen into the anoxic atmosphere by rapid mixing at the water–air interface, all added Fe(II) was completely oxidised, although at a slower rate than observed in the standing cultures (Fig. 3c), while the solubilised oxygen concentration stabilised at ~4.5 µM.

### Toxicity of green rust

As in both previously described experiments, a strong reduction in growth was observed which coincided with the formation of GR. Experiments were therefore conducted to elucidate if GR is the cause of the cessation of biological activity, or merely a secondary by-product. Therefore, cultures of *Pseudanabaena* sp. PCC7367 were set up in the anoxic, open-culture system and their growth followed by Chl *a* measurements. One set of cultures had 600 µM Fe(II) added at the start of the experiment, which would lead to the formation of GR as observed in the open-culturing experiment. At the same time the GR formation was observed in these cultures, GR was added to a parallel set of cultures during the dark cycle at levels representing the GR levels observed in the section ‘Toxicity assessment of green rust’.

**Fig. 4:**
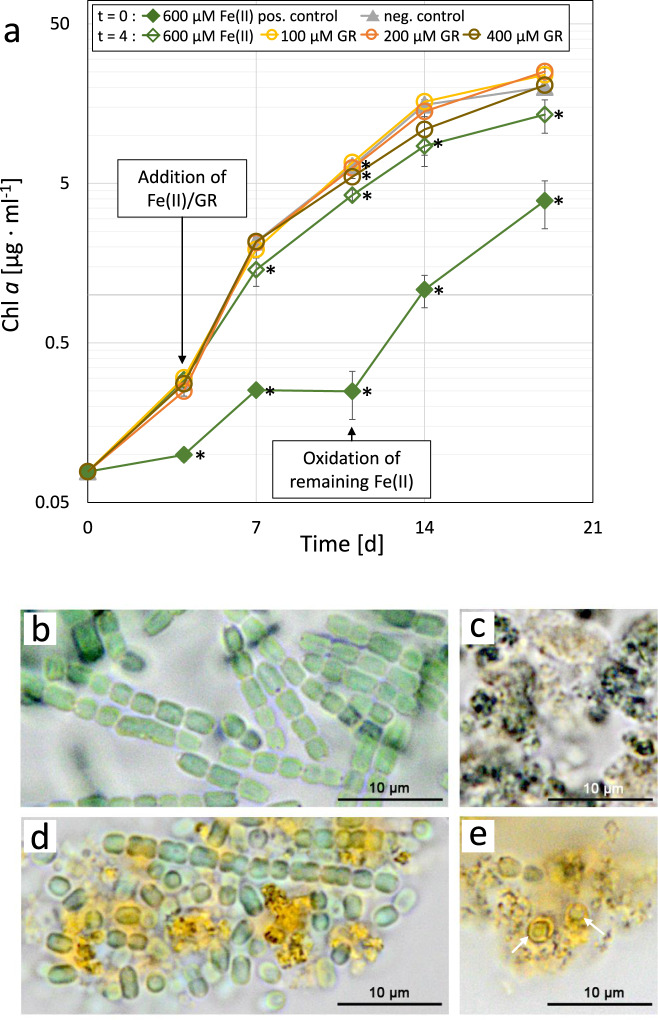
Illustration of the effect of green rust on the growth of *Pseudanabaena* sp. PCC7367. The Chl *a* growth (**a**) and light microscopic pictures (**b**, **d**, **e**) of *Pseudanabaena* sp. PCC7367 cultures after the addition of Fe(II) or green rust (GR, **c**). Chl *a* growth curves (**a**) of cultures in an anoxic open-culture system with the addition of 600 µM Fe(II) (open, green), 100 µM GR (open, yellow), 200 µM GR (open, orange) or 400 µM GR (open brown) on day 4 of exponential growth. The positive control had 600 µM Fe(II) added at the start of the experiment (closed, green), while the negative control had no addition of Fe (closed, grey). On day 6, 2 days after the addition of Fe(II)/GR, light microscopic pictures were taken of the negative control culture (**b**), the GR in an abiotic control (**c**), the culture with the addition of 400 µM GR on day 4 (**d**) and the positive control with 600 µM Fe(II) at the start of the experiment (**e**). On day 9, the remaining Fe(II)/GR in the positive control was oxidised by exposure to oxic ambient air for 10 min. Data are presented as mean values ± SD with *n* = 3 biologically independent samples. Light microscopic images were taken at 100x magnification and are presented with a 10 µm scale bar. Asterisks (*) represent a significant difference between Fe(II)/GR supplemented cultures and the growth control (*p* < 0.05, Student’s *t*-test, two-tailed).

The Chl *a* growth curve (Fig. 4a) showed that the addition of GR on day 4 had no significant effect on growth in comparison to the negative control, regardless of concentrations used. The addition of Fe(II) on day 4 showed a small but significant growth retardation of the control culture. In contrast, the positive control, with Fe(II) added to a concentration of 600 µM at the start of the experiment, showed a complete arrest of growth between days 4 and 7 until day 9. On day 9, growth could be restored by oxidising all remaining Fe(II) and GR to Fe(III) by the addition of ambient air for 10 min (Fig. 4a). Light microscopic pictures taken 2 days after the addition of Fe(II)/GR on day 6, showed intact filaments of Cyanobacteria attached to granules of yellow material with more or less dark green spots (Fig. 4d), relative to the amount of GR added 2 days prior. The dark green spots resemble the appearance of the abiotically produced GR (Fig. 4c), while the larger yellowish granules could be composed of Fe(III) compounds. The Cyanobacterial filaments and cells looked intact and appeared similar to the control culture without the addition of iron (Fig. 4b). The positive control cultures where GR was formed after Fe(II) addition, showed a similar yellowish granule structure with dark green spots (Fig. 4e) as seen in the samples where GR was added directly, however, very few intact Cyanobacteria cells were observed. In addition, structures resembling single Cyanobacteria cells encased in Fe(III) compounds (Fig. 4e, white arrows) were observed.

### Oxygen production under anoxic conditions

As the production of oxygen by marine Cyanobacteria during the Archaean was likely the main driver of the GOE, the influence of an anoxic atmosphere on the photosynthetic production of oxygen was investigated.

**Fig. 5:**
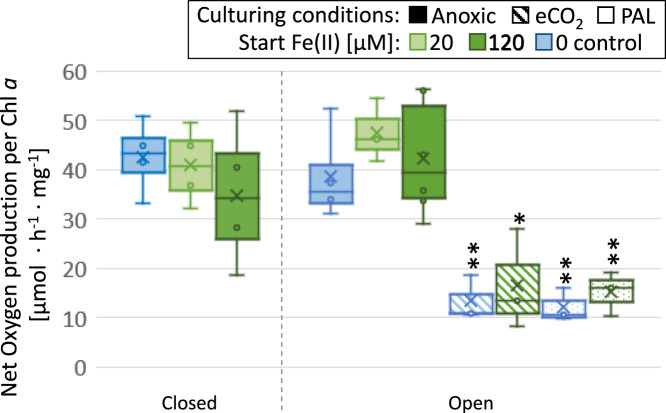
Boxplot of the hourly net oxygen production rates of *Pseudanabaena* sp. PCC7367 per unit of cellular Chl *a* content when grown under different atmospheres. The anoxic atmospheres (solid colour) tested were either N_2_ with 10% CO_2_ (closed, left) or 0.2% CO_2_ (open, right). The different Fe(II) starting concentrations were 20 µM Fe(II) (light green), 120 µM Fe(II) (dark green) or none for the control (blue). The control atmospheres were ambient air either with the present-day atmospheric levels of CO_2_ (PAL, dotted) or supplemented with 0.2% CO_2_ (eCO_2_, striped). Oxygen production was determined by measuring the oxygen accumulation in 8-day old cultures (open system and ambient air) and in 5-day old cultures (closed system) with an oxygen sensor in a cuvette and piercing oxygen needle sensor in the closed growth vessel, respectively. The light intensity during the measurement was set at the growth light intensity of 25 PPFD. Asterisks (*) represent a significant difference to the open anoxic system under the same light intensity (*p < 0.05; **p < 0.01; Student’s *t*-test, two-tailed). In the boxplot, the cross indicates the mean, the centre line shows the median value and the bounds of the box indicate the 75^th^ and 25^th^ percentiles, calculated with inclusive median, while the whiskers represent the furthest minimum and maximum measurement outliers. *n* = 4 (anoxic, closed), *n* = 3 (PAL and eCO_2_). The anoxic, open measurements (*n* = 6) had data excluded from the control and 20 µM Fe(II), which had no O_2_ production, resulting in *n* = 4 (control) and *n* = 3 (20 µM Fe(II)). All measurements were performed on biologically independent samples.

*Pseudanabaena* sp. PCC7367, grown under anoxic conditions, showed a significant (*p* < 0.05) increase in net oxygen production per mg Chl *a* in comparison to the cultures grown under modern O_2_ levels or elevated CO_2_ under the standard growth light intensity of 25 Photosynthetic Photon Flux Density (PPFD, µmol photons per m^2^/s) (Fig. 5). Measurements at 60 PPFD, which resembles 80% of the light intensity of highest measured net O_2_ production (Supplementary Fig. 8), still showed a significantly higher net O_2_ production under anoxic conditions over modern atmospheric growth conditions. The addition of Fe(II) at the start of the experiment, which was oxidised within 2 days, had no lasting, significant effect on the observed oxygen production rates, regardless of the test atmosphere.

## Discussion

Previous studies have proposed that Fe(II) at concentrations thought to have existed in the Archaean ocean were toxic to modern-day marine Cyanobacteria, when grown in a closed system with a single addition of Fe(II)^12^. This toxicity was thought to explain the delay in expansion of Cyanobacteria into the open ocean and initiation of the GOE. Our study used two of the most basal, or phylogenetically deeply rooted marine strains of Cyanobacteria to investigate the effects of Fe(II) toxicity during the late Archaean. Our data demonstrate that both Cyanobacterial strains still grow well, albeit with two-fold the doubling times of the control cultures (Fig. 3b), if supplemented with a single dose of Fe(II) at the proposed Archaean ocean concentration of 120 µM Fe(II)^23^. The single time exposure to Fe(II) did not lead to a significant alteration in net oxygen production after it was oxidised to Fe(III), neither in the open anoxic, nor in the closed anoxic cultures (Fig. 5). Additionally, net oxygen production rates under anoxic conditions in both culture systems are significantly higher than in control cultures grown under the modern-day oxygen-rich atmosphere (Fig. 5). This increase in O_2_ release in the anoxic cultures is not due to increased CO_2_ availability, since oxic control cultures with elevated CO_2_ did not produce significantly more O_2_. This experimental result contrasts with the modelled proposed reduction of 36% in O_2_ release if toxic Fe(II) at 25 µM is brought to the surface waters^12^, in that we observe the release of O_2_ during the daytime even with the addition of 120 µM Fe(II) at night (Fig. 3d), comparable to control cultures without Fe(II) addition. It is therefore unlikely that the toxicity of Fe(II) at 120 µM would have significantly reduced O_2_ production rates. Significantly, the ~4.5 µM concentration of dissolved O_2_ measured during the daylight period, in rapidly stirred cultures of *Pseudanabaena* sp. PCC7367 supplemented with Fe(II) at 120 µM at night, simulating the tidal zone, is remarkably close to the modelled 10 µM, calculated previously for oxygen oases^14^. It could also be shown that GR can form from partially oxidised Fe(II) in the open-culture growth media and its formation process could have negatively impacted Cyanobacterial growth (Fig. 4).

Our data suggest that experiments in closed systems with a single injection of CO_2_ in the headspace are not suited to investigate the influence of the proposed Archaean environmental conditions on Cyanobacterial growth and oxygen production^12^. Both strains chosen for this study show significantly longer doubling times if incubated in a closed-culture system in comparison to an open-culture system (Fig. 2a, b) or current atmospheric air controls. This is especially evident in the case of *Synechococcus* PCC7336, which exhibited almost no biological activity in the closed growth system. This reduced growth is already observed prior to when the build-up of oxygen surpasses the background oxygen concentration of the ambient air control cultures at day 8 (Supplementary Fig. 10a). Therefore, the build-up of oxygen observed over the course of the experiment is unlikely to be the cause for the initial reduction in growth. Increased photorespiration under the present atmospheric oxygen levels could be responsible for the observed differences in oxygen production rates between anoxic and oxic growth systems (Fig. 5).

This leaves only the different ambient concentrations of CO_2_ as a possible explanation for the difference in Chl *a* growth between the open- and closed-culture systems, as all other parameters are identical. In the closed-culture system, an atmosphere with 10% CO_2_ is initially used to provide CO_2_ for the whole growth cycle, while the open-growth system permits constant gaseous exchange and maintenance of CO_2_ levels at 0.2%. The slower growth at 10% CO_2_ is not caused by the small drop of pH in the buffered media from pH 7.2 to ~pH 7 due to higher levels of dissolved CO_2_ (CO_2(aq)_) (Supplementary Table 2). However, the increased CO_2(aq)_ concentration could play a major role in growth inhibition, as screening for genes associated with CO_2_ uptake revealed *Synechococcus* PCC7336 lacks the *pxcA* gene (Supplementary Table 3). The PxcA-dependent proton exchange system mediates the cellular pH homoeostasis by extrusion of protons from the cell during the uptake of CO_2(aq)_, in which CO_2_ gets converted to HCO_3_^−^ and a proton intracellularly^27^. This prevents the acidification of the cytoplasm, as PxcA-independent proton exchange systems are not sufficient to regulate the cellular pH during CO_2_ uptake below a media pH of 8^28^. Under present atmospheric levels of CO_2_, the preferential uptake of CO_2(aq)_ lasts only for seconds before the small pool of CO_2(aq)_ is exhausted and HCO_3_^−^ becomes the major source of inorganic carbon^29^. But, with the much larger pool of CO_2(aq)_ under 10% CO_2_ conditions in the closed system, the prolonged uptake of CO_2(aq)_ and subsequent internal acidification could be a major stressor in *Synechococcus* PCC7336 during growth. Even though *Pseudanabaena* sp. PCC7367 possesses the *pxcA* gene, its growth was still reduced under 10% CO_2_ conditions. Cyanobacteria possess a carbon concentrating mechanism whereby the levels of CO_2_ are raised around the enveloped enzyme Ribulose-1,5-bisphosphate carboxylase-oxygenase (RuBisCO) in the carboxysome^29^. Possibly, the CO_2_ impermeable carboxysome shell acts as a kinetic barrier for the fixation of carbon at CO_2_ concentrations larger than 3% by keeping the CO_2_ on the outside of the carboxysome, rather than concentrating it around the carbon fixing RuBisCO on the inside^30^.

The O_2_ production rates obtained in this study can now be taken into consideration when modelling the flux of atmospheric oxygen under Archaean conditions, as a higher primary-productivity rate per Chl *a* content would require less biomass and a lower nutrient influx to achieve the same level of oxygen released.

The data from the closed-culture experiment seem to confirm the previously described toxicity of Fe(II) to Cyanobacteria^12^. Concentrations of Fe(II) ≥ 120 µM lead to a significant decrease in Chl *a* growth in *Pseudanabaena* sp. PCC7367, whereas *Synechococcus* PCC7336 was unable to grow in this culture system and hence the toxic effects of Fe(II) could not be assessed (Fig. 2a, b). This was in stark contrast to the results from the open-culture system, where the addition of Fe(II) up to 120 µM led to only a short delay in the growth of *Pseudanabaena* sp. PCC7367, while *Synechococcus* PCC7336 showed a long-term reduced Chl a growth when compared to the non-supplemented controls, but with no clear dose-dependent effect. Addition of Fe(II) to a concentration of 600 µM in the open-culture system lead to the precipitation of a green precipitate within 4 days in both strains. ^57^Fe Mössbauer spectroscopy presented a spectrum that had hyperfine parameters similar to GR, although the additional presence of short-range ordered Fe(III) could not be ruled out (Supplementary Fig. 7 and Supplementary Table 4). This coincided with the arrest of all biological activity, as reflected in a lack of Fe(II) oxidation and no further increase in Chl *a* levels in the cultures (Fig. 2). A green precipitate, which we consider to be GR, also formed in the closed-culture system when the atmospheric composition was changed to a lower CO_2_ concentration of 0.2% (Supplementary Table 1). The suppression of GR formation at 10% atmospheric CO_2_ could be caused by the lower production of OH^−^ ions^27^ during the Fe(II) oxidation in the early stages of growth. The growth media is buffered to a pH between 6.9 (10% CO_2_) and pH 7 (0.2% CO_2_), but GR formation typically only occurs above pH 7.1^17^. At low concentrations of CO_2(aq)_, Cyanobacteria switch from the uptake of CO_2(aq)_ to the uptake of HCO_3_^−^ as an inorganic carbon source which releases OH^−^ ions^29, 31^. Therefore, high CO_2(aq)_ concentrations, as expected of a 10% CO_2_ atmosphere, could have delayed the switch to HCO_3_^−^ uptake and thus OH^−^ release required to raise the pH to the level required for GR precipitation. By the time the Cyanobacteria do switch to HCO_3_^−^ and start to produce OH^−^, insufficient Fe(II) remained for the precipitation of GR. These results indicate that the CO_2_ atmospheric levels might play a crucial role in the formation of GR in a neutral Archaean ocean.

As all available Fe(II) in the experiments at the proposed Archaean ocean concentration was oxidised within 2 days, the possibility of longer repeated exposure to Fe(II) was explored. Our data showed that the oxidation of Fe(II) only occurred passively by the photosynthetically produced O_2_ during the light (day) cycle, while dark (nocturnal) Fe(II) levels remain stable (Fig. 3d). Stirring the cultures to inhibit the build-up of O_2_ and slow the oxidation of Fe(II) was also explored. Unlike the incomplete oxidation of Fe(II) in previous experiments at 0.3% CO_2_^20^, all Fe(II) was completely oxidised during the light cycle at 0.2% CO_2_. However, the oxidation rate of the Fe(II) could be significantly reduced by 36 ± 15% by stirring the culture media in comparison to the non-stirred culture (Fig. 3c). Even with mixing, oxygen levels in the media remained steady at ~4.5 µM, which is in good agreement with modelled Archaean oxygen oases’ dissolved O_2_ concentrations of 1–10 µM^14^. Nevertheless, stirring only prolonged the light cycle exposure to Fe(II) by 10–30 min, depending on the photosynthetic activity of the culture. In order to reduce the Fe(II) oxidation by more than 36%, stronger agitation would be required which would most likely introduce additional shearing stress during a long-term experiment. This is supported by the observation that even a strong single daily shaking event in the continuous Fe(II) exposure experiment leads to a decrease in growth in the control cultures in comparison to previous experiments (Fig. 3b vs Fig. 2a, b). The repeated addition of Fe(II) overnight for 12 cycles caused a significant reduction in the accumulation of Chl *a* in both strains (Fig. 3b). During the light cycle, all Fe(II) was rapidly oxidised, which suggests that Cyanobacterial mats could indeed form oxygen oases, as suggested by Farquhar et al.^32^, shielding them from the influence of Fe(II)^12^.

Oxygen oases could also have released oxygen to the atmosphere locally, as the measured iron oxidation rates are only 16–25% of the net oxygen production rates on a per mol basis (Figs. 3d and 5). This is supported by evidence from the rock record which show mild oxidative weathering of surface rocks releasing important micronutrients like molybdenum^18^. In the shallow-water coastal regions, the produced Fe(III) could have reacted with the tidal influx of a ferruginous Archaean ocean to form GR, as was observed during the nightly additions of Fe(II). This may also be the case in the open ocean, with upwelling of Fe(II) in the water column reacting with Fe(III) produced during the day to form GR. In an Archaean ocean environment, the produced GR (or Fe(III) precursor) may have been washed into the open ocean and contributed to the formation of BIF as illustrated in Fig. 1^17^. Experiments where GR was added directly to exponentially growing cultures of *Pseudanabaena* sp. PCC7367 showed that pure GR had no significant effect on growth, even at concentrations four-fold higher than the amounts obtained in the open-culturing experiment (Fig. 4a). Most likely, the formation process of GR/Fe(III) hydroxides caused the inhibition of the growth of *Pseudanabaena* sp. PCC7367, as the complete oxidation of remaining media Fe(II) and GR precipitate restored growth (Fig. 4a). Light microscopic images show that the oxidation of Fe(II) in the media by *Pseudanabaena* sp. PCC7367 lead to the encrustation of cells by the oxidation products (Fig. 4d), whereas the addition of GR resulted in cells adhering to the outside of the GR granule (Fig. 4e) without inhibition of growth (Fig. 4a) or change in growth morphology, as compared to the control (Fig. 4b). Therefore, our data suggest that only the formation of GR, not GR itself, had an inhibitory effect on growth (Fig. 4), which could be overcome in cultures with sufficiently high oxygen production to completely oxidise all media Fe(II), thereby preventing their encrustation by newly formed GR/Fe(III) hydroxides (Figs. 3a and  4a). Otherwise, the ongoing formation of GR would lead to a self-reinforcing process, whereby the decreasing photosynthetic activity by GR formation prevents the detoxification of Fe(II) by oxidation until the culture enters a state of minimal biological activity. A possible mode of GR toxicity could be the potential mechanical stress during the formation of GR on already Fe(III) encrusted bacterial cells. Therefore, we propose that the widespread colonisation of Archaean shallow-water environments by Cyanobacteria, specifically the multicellular, benthic *Pseudanabaena* sp. PCC7367 ancestral strain used in this study, was not delayed by the toxicity of Fe(II), or by the formation of GR under the levels of Fe(II) thought to have been present in the Archaean ocean. Additionally, our study emphasises the importance of experimental setup, diurnal cycling as well as strain selection (unicellular vs multicellular; benthic vs planktonic) and growth optimisation, in order to gain insight into the events leading up to the oxygenation of the Earth’s atmosphere.

## Methods

### Cultures

*Pseudanabaena* sp. PCC7367 and *Synechococcus* sp. PCC7336 were purchased from the Pasteur Culture Collection (PCC) and maintained in ASN-III medium (PPC^33^) on a 16:8 h light–dark cycle in a Percival culture chamber (E-22L) at 24 °C, 65% relative humidity under normal atmospheric composition (400 ± 20 ppm CO_2_). Both cultures were grown at 25 PPFD (µmol photons per m^2^/s), the highest light intensity shown not to induce light stress defined by not increasing the carotenoid (CA) over Chl *a* ratio (Supplementary Fig. 9). Light intensity in PPFD was measured with a Li-Cor Quantum sensor attached to the Li-250A Light Meter (Li-Cor, USA). Cultures grown under anoxic conditions were maintained in ventilated T_75_ suspension culture flasks (Sarstedt, Germany) in a self-built anoxic box (construction plans and software available at biorxiv.org/cgi/content/short/2020.12.17.423238v1), which was placed inside a culture chamber (Percival E-22L) with the same light and temperature settings as the control cultures. An anoxic atmosphere was retained by constantly removing oxygen generated during photosynthesis with forming gas (Arcal F5, AirLiquide, Germany) on a palladium catalyst (NeoLab Migge, Germany), while 0.2% of CO_2_ was maintained by a WMA-4 CO_2_ Analyzer (PP-Systems, USA). Forming gas and N_2_ inflow as well as O_2_, H_2_ and relative humidity levels were regulated with an Arduino-based control system (Seeeduino lotus, Seeed studio, China).O_2_ levels (±6 ppm) were monitored using a ME2-O2-Ф20 oxygen sensor (Zhengzhou Winsen Electronics, China). Both strains were acclimated to their culture conditions for at least 8 weeks (4 transfers) prior to commencing the initial growth curve experiments. A repeat growth curve assessment 6 months later in the anoxic growth chamber Glovebox Mega E-Line 4 (Glovebox Systemtechnik, Germany) did not show any difference in doubling times compared to the self-built anoxic box. The Glovebox anoxic growth chamber uses a N_2_ atmosphere without added hydrogen, where O_2_ is constantly removed by a regeneratable copper catalyst. All other environmental parameters, such as CO_2_ concentration or lighting regime and intensity were exactly replicated as in the self-built anoxic box. O_2_ levels (±0.1 ppm) in the anoxic growth chamber were monitored using an AutoOxi R 2.01 oxygen trace sensor (Glovebox Systemtechnik, Germany). All experiments that required an anoxic atmosphere after the initial comparison of open-culturing vs closed-culturing growth systems were conducted in the more spacious Glovebox anoxic growth chamber.

### Comparison of growth systems

For the closed-culture system, 80 ml of ferric ammonium citrate-free ASN-III media, buffered with 22 mM NaHCO_3_, was dispensed into 100 ml acid-washed, laboratory glass bottles (Schott, Germany) and sealed with butyl rubber stoppers (Zscheile & Klinger, Germany). The control cultures were set up in standard ASN-III media, buffered with 22 mM NaHCO_3_.

Sealed media aliquots were made anoxic by gassing for 20 min with N_2_ gas (99.99%) before transferring them to the anoxic box for inoculation and Fe(II) addition. Fe(II) was added to a final concentration of 20, 120 or 600 µM in the form of an anoxic, sterile filtered 0.2 M FeCl_2_ solution in ddH_2_O and the concentrations verified by means of the spectrophotometric ferrozine-based assay. *Pseudanabaena* sp. PCC7367 and *Synechococcus* PCC7336 were acclimatised to the anoxic growth conditions for between 2 and 7 months, depending on the experiment conducted. Cultures were inoculated with exponentially growing starter cultures to a final Chl *a* concentration of 0.1 µg/ml. After inoculation the bottles were sealed, removed from the anoxic box and gassed for 15 min with Process CO_2_/H_2_/N_2_ 5/10/85 (AirLiquide, Germany). The culture vessels were placed on their sides to maximise light and atmospheric exposure in the culture box for 21 days.

In setting up the open-culture system, media was aliquoted into ventilated T_75_ suspension culture flasks (Sarstedt, Germany), transferred into the anoxic box and equilibrated by daily shaking with the anoxic atmosphere (N_2_; 5% H_2_; 0.2% CO_2,_ ~1 ppm O_2_) for 1 week prior to inoculation. The inoculation and addition of Fe(II) to this open-culture system was conducted similarly to the closed system experiment. Cultures were incubated within the anoxic box for 21 days at the growth conditions described above.

Cyanobacterial growth and Fe(II) oxidation in both experimental setups were monitored by Chl *a* and ferrous iron determinations for 21 days, respectively. The evolution of oxygen inside the closed growth vessels was measured using an OX-NP oxygen needle sensor (UNISENSE, Aarhus, Denmark) with a detection limit of 0.3 µM dissolved oxygen, which was calibrated according to the manufacturer’s instructions. One sensor was permanently inserted in one control culture of *Pseudanabaena* sp. PCC7367 in buffered ASN-III media for constant monitoring of the dissolved O_2_ and logged its concentration every 30 s with the Unisense SensorTrace Suite software (v3.2.000). Dissolved O_2_ was measured in all closed cultures 10 min before the start of the light cycle and 5 h after the lights went on, on days 3, 5, 6, 8, 14, 15 and 20.

### Chlorophyll *a* and carotenoid determination

Chl *a* and CA were extracted in 90% (v/v) neutralised methanol and measured as described in Herrmann and Gehringer^34^. The absorption at 780 nm was used for baseline corrections.

### Spectrophotometric ferrozine iron assay

The spectrophotometric ferrozine assay was used to determine the amount of Fe(II) in the cultures and track its oxidation to Fe(III). Ferrozine (Disodium-4-[3-pyridin-2-yl-6-(4-sulfonatophenyl)-1,2,4-triazin-5-yl]benzosulfonate) is a bidentate ligand of metal ions, which binds with Fe(II) forming a brightly purple coloured complex at 562 nm^35^. Samples inside the anoxic chamber were taken by drawing 500 µl of media and precipitating non-soluble components by centrifugation for 1 min at 15,000*g*. Samples from the closed-culture experiments were drawn off with a syringe and immediately added to 1 volume of 2 M HCl to prevent oxidation during the subsequent 1 min 15,000*g* centrifugation step. The following assay was prepared and conducted as described by Viollier et al.^36^. The total reaction volume was reduced to 50 µl ferrozine solution (50% w/v Ammonium acetate, 0.1% w/v Ferrozine in ddH_2_O) with 200 µl sample in order to measure sample absorption at 560 nm in a 96-well plate reader (Multiscan FC, ThermoFisher Scientific, USA). Fe(II) calibration solutions were made up fresh from FeSO_4_ in 1 M HCl. Calibration solutions for total iron determination were prepared by dissolution of high-purity Fe powder ( >99.9%) in 6 M HCl. In order to determine the total concentration of iron, samples of shaken cultures were added to 1 volume of 2 M HCl to solubilise finely precipitated solid Fe compounds. Fe(III) was then reduced to Fe(II) by the addition of 50 µl 10% (w/v) hydroxyl hydrochloride in 1 M HCl to 200 µl sample and incubated for 30 min in a 96-well plate at room temperature prior to the addition of 50 µl ferrozine solution.

### Fe(II) retention and continuous exposure

In order to assess the amount of Fe(II) oxidation during the dark cycle, 7-day old, exponentially growing cultures of *Pseudanabaena* sp. PCC7367 and *Synechococcus* PCC7336 were used. Cultures and media were prepared and inoculated as for the open-culture experiment and supplemented with 120 µM Fe(II) at the start of the experiment. The cultures were maintained anoxically in a Glovebox Mega E-Line 4 (Glovebox Systemtechnik, Germany) at 25 PPFD, 24 °C, 65% humidity, 0.2% CO_2_ and ≤1 ppm O_2_ on an 18:6 h light–dark cycle. On day 7, the cultures were made anoxic by shaking them for 45 min after the start of the dark cycle and measuring the remaining dissolved oxygen concentration (OX-NP sensor) and Fe(II) levels (ferrozine assay). Sterile 0.2 M FeCl_2_ solution was added to a final concentration of 120 µM and verified by a second ferrozine assay at 5 min after addition. An OX-NP sensor permanently monitored the dissolved oxygen in one culture flask of each strain. The Fe(II) concentration was measured periodically during the dark cycle and every 10 min after the start of the light cycle, until no more Fe(II) was detectable, as well as a final measurement of the total Fe inside the media 150 min after the lights turned on. As Fe(II) levels remained nearly constant during the dark cycle, this method was used to evaluate the potential toxicity of repeated exposure to Fe(II), at levels approaching that of the Archaean ocean. *Pseudanabaena* sp. PCC7367 and *Synechococcus* PCC7336 were set up as described for the open-culturing system and supplemented every dark cycle to 120 µM Fe(II). The influence of Fe(II) on growth was tracked by daily Chl *a* determinations.

The influence of wave mixing on the oxidation of Fe(II) and the release of oxygen was assessed in a similar manner to the overnight retention experiments. A sterile magnetic stirring bar (442-0487, VWR, USA) was placed into 7-day old exponentially growing cultures of *Pseudanabaena* sp. PCC7367 during the day cycle and the growth vessel placed on a magnetic stirrer (AAN1.1, Roth, Germany) at 250 rpm. After the medium O_2_ concentration got stabilised, Fe(II) was added to a concentration of 120 µM and its oxidation was determined every 10 min by the ferrozine assay.

### Toxicity assessment of green rust

In order to determine the potential toxicity of the GR observed in the open-culture experiments initially provided with 600 µM Fe(II), cultures of *Pseudanabaena* sp. PCC7367 were exposed to different levels of GR during exponential growth. Cultures were prepared and inoculated as for the open-culture system experiment. Abiotically produced GR was added on day 4 to a total concentration of either 100, 200 or 400 µM, corresponding to the maximum GR levels that would be produced by the addition of Fe(II) at 600 µM, 1.2 mM or 2.4 mM, respectively. Care was taken to ensure that no unbound Fe(II) was added with the GR. Positive controls were made by adding sterile FeCl_2_ solution to a concentration of 600 µM to cultures during inoculation, or in parallel to the addition of GR on day 4. Cultures without the addition of GR or Fe(II) were used as negative controls. The growth was followed by regular Chl *a* measurements. Two days after the addition of GR, light microscopic pictures were taken at 100x magnification (Olympus BX53, Japan). On day 9, the Fe(II) and GR in the positive controls were oxidised by removing the cultures from the anoxic chamber followed by gentle shaking under ambient air for 10 min before being reinserted into the anoxic chamber. Abiotic GR was produced by anoxically adding FeCl_2_ to 50 ml of 22 mM NaHCO_3_-buffered ASN-III media to a total concentration of 15 mM and adjusting the medium pH to 8 through the addition of 0.1 M KOH. This solution was exposed to ambient air while shaking until a shift in colour from initially translucent green to turbid yellow, and finally turbid dark green was observed. The dark green precipitate was collected after centrifugation at 12,000*g* for 10 min (Centrifuge 5810 R, Eppendorf, Germany) and two subsequent washing steps with anoxic, buffered ASN-III to remove all unreacted Fe(II). The pellet was suspended in 5 ml buffered ASN-III media and the concentration of the GR as well as its Fe(II)/Fe(III) ratio was determined by the spectrometric ferrozine assay.

### Net oxygen production rates

In order to calculate the net oxygen production rates under different atmospheric conditions, the dissolved oxygen evolution over time was measured by a retractable fibre oxygen minisensor (OXR430, PyroScience, Germany) connected to a MINI-PAM-II (Walz, Germany) inside a constantly stirred measurement cuvette and recorded using the WALZ Wincontrol 3 software V3.28. Cultures were set up as for the open-culture setup in T_25_ ventilated suspension flasks in buffered ASN-III with and without the addition of 120 µM Fe(II). The oxic cultures were then exposed to normal air with present-day atmospheric levels (PAL) of CO_2_ or supplemented with 0.2% CO_2_. The anoxic cultures in the open-culture system were exposed to an anoxic atmosphere with 0.2% CO_2_. Measurements were conducted on 8-day old, mid-exponential phase cultures that were shaken to equilibrate to the growth atmosphere, before 400 µl was transferred into the suspension cuvette (KS-2500, Walz, Germany) of the MINI-PAM-II, which was also situated in the corresponding atmosphere. The Chl *a* content of 1 ml of culture was determined in parallel. Oxygen measurements were taken for 5 min using the standard growth light irradiance of 25 PPFD under their experimental atmospheric conditions. The difference between the start and end point measurements/readings of dissolved oxygen and the Chl *a* concentration was used to calculate the net oxygen production rates per mg Chl *a* per hour. The net oxygen production rate in the closed anaerobic growth vessels was determined from the oxygen measurements taken on day 5 as described in the section ‘Comparison of growth systems’.

### Statistics and reproducibility

All statistical analyses were done using a two-tailed, heteroscedastic Student’s *t*-test (Excel 365, Microsoft, USA) to determine the influence of Fe(II) treatment and the effect of different growth systems on the organisms’ growth and O_2_ production levels. The exact *p* values are included in the data files for the figures. All experiments were performed once, with measurements taken in parallel from independent biological replicate samples under the same growth conditions.

### Mineralogical analysis

In order to determine the composition of the green precipitate, which was formed in the open-culture experiments with an Fe(II) concentration of 600 µM, 4 ml of shaken culture was centrifuged at 15,000*g* for 15 min and the pellet was washed twice with ddH_2_O. The dry pellets were dissolved in 2 M HCl and the ratio of Fe(II)-to-Fe(III) was determined by the ferrozine assay. In order to collect enough sample material for Mössbauer spectroscopy, 400 ml culture was set up in an open-culture system (with 600 µM Fe(II)) in acid washed, sterile Fernbach flasks with sterile gauze permitting free gaseous exchange. After 1 week, all the precipitate was collected and processed as above, with the addition of a vacuum drying step prior to ^57^Fe Mössbauer spectroscopic analysis.

The sample was prepared for ^57^Fe Mössbauer in an anoxic Glovebox (100% N_2_) within which the sample precipitate was sealed inside a plexiglas holder (diameter = 1 cm) and stored in an airtight jar at −20 °C until measurement. The sample was loaded into a closed-cycle exchange gas cryostat (Janis cryogenics) under a backflow of helium. The measurement was collected at 77 K with a constant acceleration drive system (WissEL) in transmission mode with a ^57^Co/Rh source and calibrated against a 7 µm thick α-^57^Fe foil measured at room temperature. All spectra were analysed using Recoil V1.05 (University of Ottawa) by applying a Voight-based fitting (VBF) site analysis^37^. The half-width at half-maximum (HWHM) was fixed to 0.125 mm/s during fitting.

### Growth curve

Maximum doubling times were determined as the inflection of the slope of the growth curve in the exponential phase and used to calculate the doubling time^38^.

### Gene screening

The presence of a *pxcA* gene in *Pseudanabaena* sp. PCC7367 (GCA_000317065.1) and *Synechococcus* PCC7336 (GCA_000332275.1) was tested using nBLAST and PSI-BLAST in BLAST+, version 2.10.0^39^. Sequences from *Synechocystis* sp. PCC 6803 were used as reference gen and protein sequence, respectively. The matches with the highest scores are shown in Supplementary Table 3.

### Reporting summary

Further information on research design is available in the Nature Research Reporting Summary linked to this article.

## Supplementary information

Supplementary Information

Reporting Summary

## Data Availability

Source data for figures (Figs. 2–5 and Supplementary Figs. 8–10) as well as the values for the statistic tests are available on the Zenodo data repository 10.5281/zenodo.4550675. The authors declare that all other data supporting the findings of this study are available within the paper and its supplementary information files. The gene and protein sequences used in the screen for the *pxcA* gene (Supplementary Table 3) are accessible through https://www.ncbi.nlm.nih.gov/ under the following links: 14615844, BAA16993.1, CP003592.1WP_015163468.1, AF448078 and WP_162139143. The construction plans and software for the self-designed anoxic box are available at biorxiv.org/cgi/content/short/2020.12.17.423238v1.
