# Supplementary material for: Diurnal Fe(II)/Fe(III) cycling and enhanced O2 production in a simulated Archean marine oxygen oasis
Source: Nat Commun. 2021 Apr 6;12:2069. doi: 10.1038/s41467-021-22258-1 (PMC8024245; doi:10.1038/s41467-021-22258-1)
Supplement: Supplementary file 1 — Supplementary Information [file 41467_2021_22258_MOESM1_ESM.pdf]

Supplementary information

Diurnal Fe(II)/Fe(III) cycling and enhanced O_2 production in a simulated Archean marine oxygen oasis.

Herrmann A.J.[1], Sorwat J.[2], Byrne J.M.[2], Frankenberg-Dinkel, N. [1] and Gehringer M.M.[1]*

[1]Department of Microbiology, Technische Universität Kaiserslautern, 67663 Kaiserslautern, Germany

[2]Geomicrobiology, Centre for Applied Geosciences, University of Tübingen, 72074 Tübingen, Germany

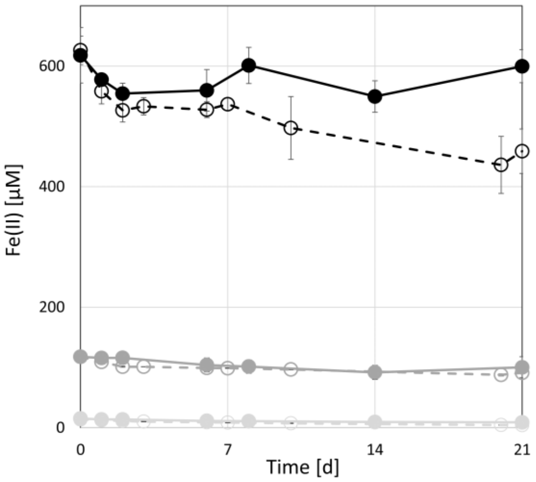

Supplementary Fig. 1: Ferrozine assay results of control media. Fe(II) levels in anoxic control media, buffered with 22 mM NAHCO3, and amended with Fe(II), was determined by ferrozine assay every two to three days for 21 days in closed anoxic culture bottles (solid lines) or open cell suspension flasks in an anoxic workstation (dashed lines). The Fe(II) starting concentrations were 20 µM (light grey), 120 µM (medium grey) and 600 µM (black). Data are presented as mean values ± SD with n = 4 biologically independent samples.

Supplementary Table 1: Average Fe(II)/Fe(III) ratio of the precipitate from 7 day old cultures of *Pseudanabaena* PCC7367 after the addition of Fe(II) to a concentration of 600 µM as determined by ferrozine assays. Cultures were grown in an open-culture system with 0.2% CO_2 or in a closed-culture system with either 0.2% or 10% CO_2. Data are presented as mean values ± SD with n = 3 biologically independent samples.

CO_2 conc. [%]	Culture system	Fe(II)/Fe(III) ratio
0.2	open	2.38 ± 0.15
0.2	closed	2.23 ± 0.07
10	closed	2.76 ± 0.24

Supplementary Table 2: Average Chl *a* concentration of 15 day old cultures of *Synechococcus* PCC7336 grown in ASNIII Media buffered at pH 6.8 and pH 7.2 and unbuffered at ~ pH 7.4. Data are presented as mean values ± SD with n = 3 biologically independent samples.

Media	pH	Chl *a*
ASNIII, unbuffered	7.4	1.42 ± 0.06
ASNIII, buffered	6.8	1.38 ± 0.04
ASNIII, buffered	7.2	1.7 ± 0.28

Supplementary Table 3: Gene screen results for *Pseudanabaena* sp. PCC7367 and *Synechococcus* PCC7336. The genomic sequences of both organisms, GCA_000317065.1 and GCA_000332275.1 respectively, were screened for the presence of a PxcA encoding gene. Shown is the best hit, as indicated by nBlast or PsiBlast. The presence or absence of the gene is indicated by a '+' or '–' for each organism.

Organism	nBlast	Cover	E-Value	PsiBlast	Cover	E-Value	Present
Synechocystis sp. PCC 6803 reference gene	14615844	-	-	BAA16993.1	-	-	
Pseudanabaena sp. PCC7367	CP003592.1 233432 to 233646	18%	4.00E-08	WP_015163468.1	97%	2.00E-65	+
Synechocococcus sp. PCC7336	AF448078	7%	2.8	WP_162139143.1	13%	0.46	-

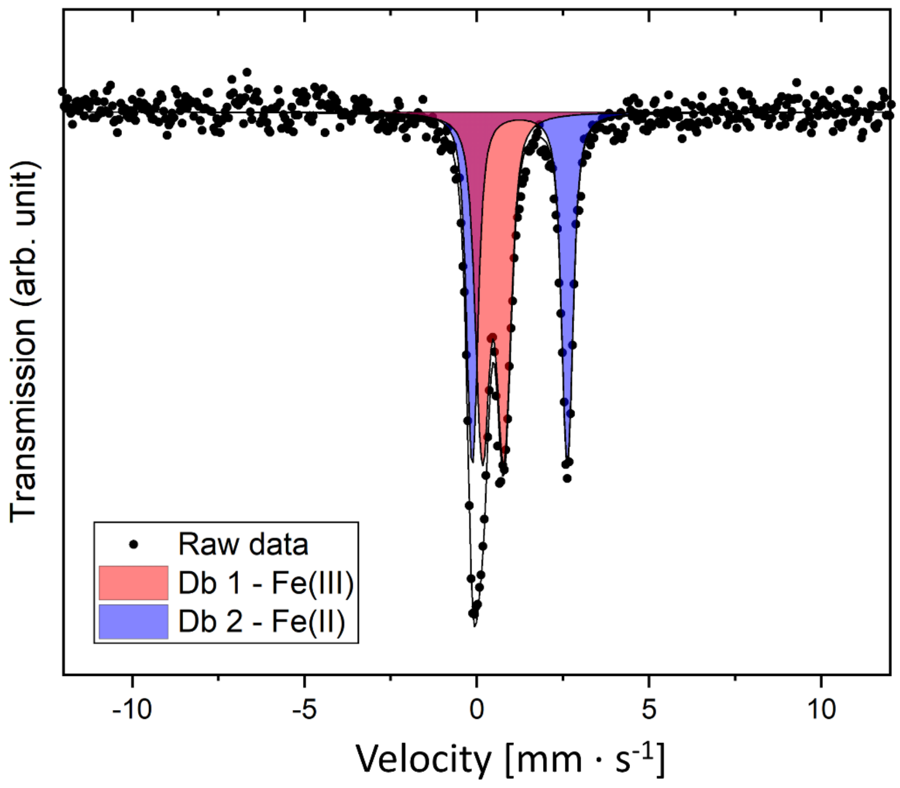

Supplementary Fig. 2: Transmission ^{57}Fe Mössbauer spectra measurement at 77 K of the green precipitate which formed in cultures with 600 µM Fe(II) in the open-culture system.

Supplementary Table 4: Mössbauer hyperfine parameters measured at 77 K of the spectra presented in **Supplementary Fig. 2**. Db – spectral doublet; Ox. State – oxidation state; CS – center shift; QS – quadrupole splitting; R.A. – relative abundance.

Site	Ox. State	CS (mm/s)	QS (mm/s)	stdev [QS] (mm/s)	skew [QS] (mm/s)	R.A. (%)	Error [R.A.] (%)
Db 1	Fe(III)	0.47	0.63	0.28	0.13	52.5	1.1
Db 2	Fe(II)	1.25	2.76	0.18	0.00	47.5	1.1

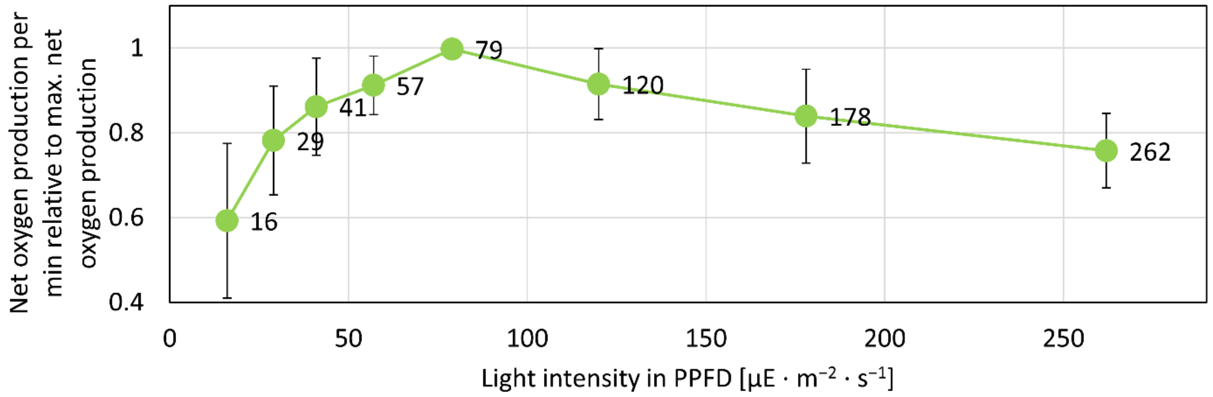

Supplementary Fig. 3: Oxygen production per min at different light intensities of exponentially growing *Pseudanabaena* sp. PCC7367 under anaerobic conditions relative to the maximum measured oxygen production value. Data are presented as mean values ± SD with n = 4 biologically independent samples.

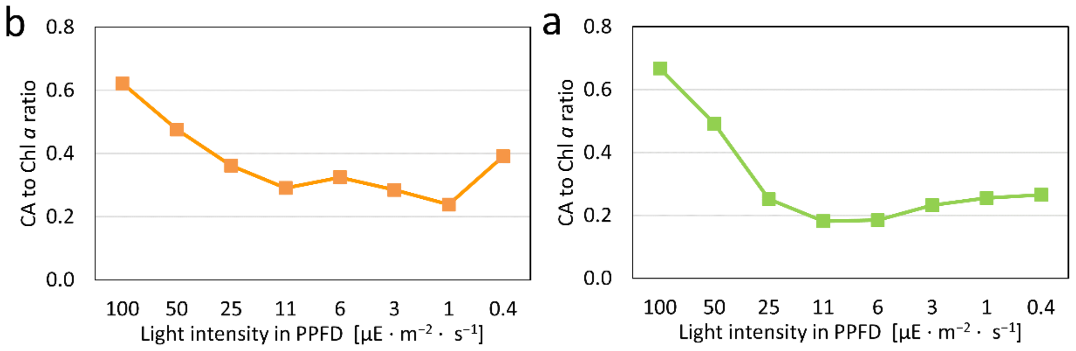

Supplementary Fig. 4: Pigment content determinations to determine optimal growth light intensity. Plots of the carotenoid (CA) to chlorophyll *a* (Chl *a*) ratios of cultures of *Pseudanabaena* sp. PCC7367 (a) and *Synechococcus* PCC7336 (b) grown under different light intensities for 14 days. n = 1

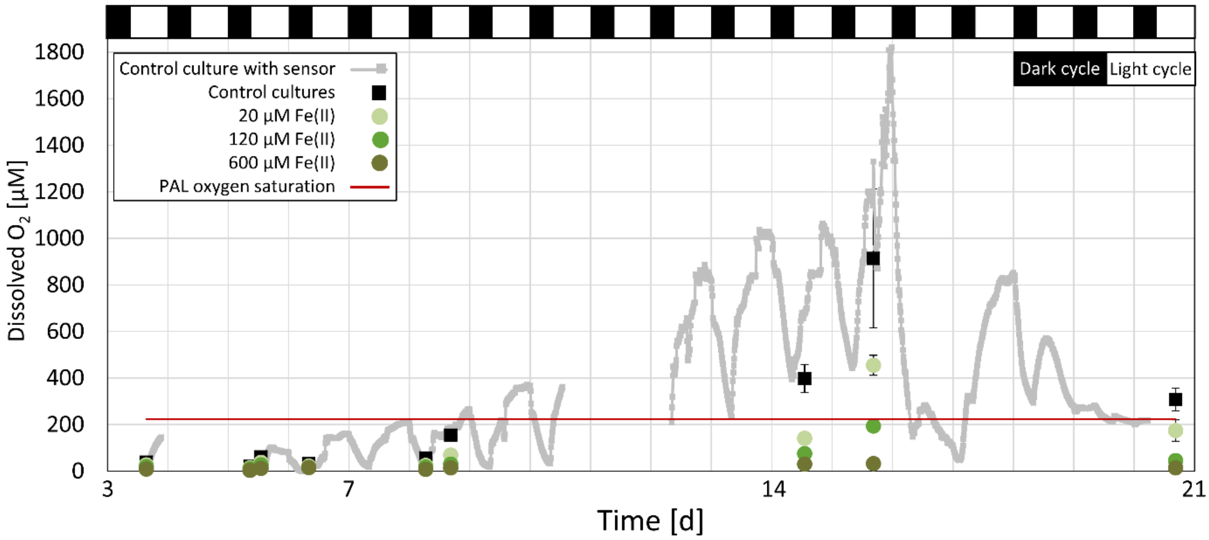

Supplementary Fig. 5: Oxygen accumulation in a closed system of *Pseudanabaena* sp. PCC7367 over the course of 21 days. During the closed culture system experiments, the dissolved oxygen levels of the culture media was assessed during different growth phases. Depicted are the mean value and standard deviation for n = 4 biologically independent samples at different starting Fe(II) concentrations (black = control; light green = 20 µM; green = 120 µM; dark green = 600 µM), as well as the dissolved oxygen levels of one control culture with a permanently inserted oxygen sensor (grey line) of *Pseudanabaena* sp. PCC7367. The red line indicates the oxygen saturation level under present day atmospheric conditions (PAL). The measurements at day five were used to calculate the oxygen production rates per mg Chl *a* per hour for the closed system (**Error! Reference source not found.**). Data for the reference culture from day 4-5 and 10-12 were not recorded because of a hardware malfunction.
